# The Experience of Cancer-Related Cognitive Impairment Across Common Cancers: Protocol for a Qualitative Systematic Review

**DOI:** 10.2196/56888

**Published:** 2024-05-31

**Authors:** Maryam Ibrar, Harleen Kaur Rai, Ashleigh Main, Haruno McCartney, Roma Maguire, Mario Alfredo Parra Rodriguez

**Affiliations:** 1 Department of Computer and Information Sciences University of Strathclyde Glasgow United Kingdom; 2 Department of Psychology University of Strathclyde Glasgow United Kingdom

**Keywords:** cancer, neoplasms, cancer survivors, cancer-related cognitive impairment, chemotherapy-related cognitive impairment, qualitative research, executive function, cognition, cognitive impairment, quality of life, common cancer, qualitative synthesis, adult, young adult, functional ability, functional outcome, qualitative, cognitive impairments, cancer survivor, survival rates

## Abstract

**Background:**

Cancer-related cognitive impairment (CRCI) is commonly experienced by patients with cancer during treatment, and 35% of patients experience cognitive impairment after treatment completion. Impairments in memory, attention, executive functioning, and information processing speed are most reported and often negatively impact daily functioning and quality of life (QoL). Despite the large scale of reports, this adverse side effect is underinvestigated across common cancer types, and there is a lack of insight into the CRCI experience.

**Objective:**

This qualitative synthesis aims to explore the evidence in relation to the experience of CRCI across common cancers. It also aims to understand the prevalence of CRCI across various cancer types, cognitive domains, and its impact on QoL and functional ability.

**Methods:**

A comprehensive search of databases, including PubMed, American Psychological Association PsycINFO, CINAHL, and Scopus, will be conducted. A total of 2 independent reviewers will screen titles and abstracts for inclusion, followed by full-text screening. A third reviewer will resolve any arising conflicts in the process of data screening and inclusion. Subsequently, data extraction and quality assessment using the Critical Appraisal Skills Programme (CASP) tool will be conducted. The results will be analyzed using thematic analysis.

**Results:**

This review is part of a PhD program funded in January 2023. The review commenced in June 2023, and data analysis is currently in progress. The qualitative synthesis will explore the experiences of CRCI across common cancers. The included studies are expected to report on numerous cancer types such as breast cancer, prostate cancer, leukemia, and lung cancer. The included study types are most likely to be interviews, focus groups, and surveys with qualitative components.

**Conclusions:**

This protocol highlights the need for a qualitative synthesis that will explore the experience of CRCI across common cancer types. It will provide valuable insight into the lived experience of CRCI and the cognitive domains that may be disproportionately affected. There is a growing demand for further management interventions and clinically tested treatments of CRCI and the qualitative exploration of patient experience is crucial for their development. This qualitative synthesis will inform future developments and will contribute to improving QoL after cancer.

**International Registered Report Identifier (IRRID):**

DERR1-10.2196/56888

## Introduction

### Background

The global cancer population is growing rapidly with an estimated 19.3 million new cases and almost 10 million deaths annually [1]. Fortunately, with advances in cancer treatment, survival rates have improved significantly. Despite their well-recognized side effects (eg, joint pain, nausea, depression, and anxiety) [2], treatments, such as chemotherapy, radiotherapy, and targeted cancer drugs, all play a major role in decreasing the incidence and risk of death [3]. Around 75% of patients experience quantifiable cognitive impairment throughout treatment and 35% go on to experience cognitive impairment posttreatment for months and years to follow [4].

Cancer survivors often report problems with memory, attention, executive functioning, and information processing speed [5]. These symptoms associated with cancer treatment are referred to as cancer-related cognitive impairment (CRCI), “chemobrain,” or “chemotherapy-induced cognitive impairment (CICI)” [6]. There is currently no standard definition or description of CRCI and the mechanism behind this phenomenon remains unknown [7]. Although not all patients will experience CRCI, some risk factors increase susceptibility such as low cognitive reserve, older age, genetics, and lifestyle factors [8]. The long-term toxic impact of cancer treatments on neurological functioning is a crucial area to investigate in terms of quality of life (QoL).

There is limited literature surrounding CRCI but from current research, we now understand the daily challenges associated with it and the negative impact on QoL [9]. Existing qualitative studies investigating the experience of CRCI in the breast cancer population discovered that patients report feeling “less sharp,” which often becomes apparent when returning to routine activities. This has an adverse impact on their ability to function in everyday life [6]. Despite increasing numbers of survivors managing to return to work, CRCI is commonly reported but is not well managed in corporate workplaces. The most reported problems involve planning and executing work and many survivors described feeling fatigued and experiencing psychological distress [10]. Remaining in employment while experiencing CRCI is associated with better QoL than resigning from employment. Therefore, developing management strategies for CRCI in the workplace is of interest to both patients and employers [11]. Furthermore, cognitive symptoms, such as language deficits, also impact interpersonal relationships by causing confusion and miscommunication between family and friends. There are also reports of family members downplaying the severity of symptoms and others report their authority being challenged due to their impairments [12].

There are currently no effective treatments for CRCI; however, there are some interventions that can be implemented to alleviate symptoms. In severe cases, pharmaceutical options, such as antidementia drugs, can be administered to manage cognitive impairment but there is limited clinical evidence of their efficacy [13]. Patients with breast cancer with CRCI were administered donepezil, an antidementia drug, in a trial that found no significant improvements in memory and other cognitive functions [14]. In most cases, these drugs are not required and various management techniques, such as physical exercise, mindfulness, and cognitive training, are implemented [15]. Engaging in aerobic exercise and strength training over a period of 6 months has been shown to improve self-reported cognitive issues, fatigue, and QoL [16]. However, physical exercise may not be feasible for all patients with cancer as treatment side effects often include chronic illnesses such as joint pain [2]. Management techniques often involve introducing prompts such as setting alarms, making notes, writing lists, and implementing a healthy diet can also help. Nonetheless, there is a growing need and demand for alternative evidence-based CRCI treatment and management from patients [17].

Despite the large scale of reports, this adverse side effect is underinvestigated across common cancer types and there is a lack of insight into the CRCI experience. There is insufficient qualitative evidence relating to the experience of CRCI. Existing research primarily focuses on breast cancer compared with other cancers which are often overlooked (eg, prostate, lung, colorectal, and kidney). CRCI is a serious difficulty that has negative implications on daily functioning and QoL and there is currently no sufficient intervention. This review will aim to fill the gap in this field by exploring qualitative and mixed method evidence of the CRCI experience across common cancers. Such a review has not been previously conducted. The findings will provide insights into the prevalence of CRCI, consolidate current knowledge, and identify gaps in the research area. The outcomes could also define specific cognitive domains, cancer types, or treatments that may be disproportionately affected. This review will inform further developments of resources to support the maintenance and management of CRCI.

### Objectives

This qualitative synthesis aims to (1) explore the qualitative evidence in relation to the experience of CRCI across common cancers, (2) understand the prevalence of CRCI across various cancer types, (3) understand the impact of CRCI on QoL and functional ability, and (4) understand the impacted cognitive domains.

The research question is as follows: “What are cancer survivors’ experiences of cancer-related cognitive impairment across common cancers?”

## Methods

### Design

The data collection and analysis of this qualitative synthesis will be conducted and reported in compliance with the PRISMA (Preferred Reporting Items for Systematic Reviews and Meta-analyses) [18] and will adhere to the “Enhancing Transparency in Reporting the Synthesis of Qualitative Research” (ENTREQ guidelines) [19].

### Eligibility Criteria

The population phenomenon of interest and context (PICo) framework will be applied to evaluate studies and guide inclusion and exclusion criteria (Textbox 1) [20].

**Eligibility criteria.**

**Inclusion criteria**

- Interviews
- Focus groups
- Only qualitative components of mixed method surveys including verbatim quotes
- Qualitative data exploring patient experience of cancer-related cognitive impairment
- Qualitative data extracted from mixed method studies

**Exclusion criteria**

- Gray literature
- Reviews
- Quantitative data including questionnaires with scales or any data without verbatim quotes
- The experience or perception of caregiver or practitioners
- Conditions other than cancer
- Cases where there is no mention of cognitive impairment

### Population

The qualitative synthesis will include studies involving patients aged 18 years or older, who are currently undergoing or have completed any cancer treatment and experienced cognitive impairment. There will be no restriction on gender, tumor type, or comorbidities.

### Phenomenon of Interest

The phenomenon of interest in this protocol is the experience of CRCI. As there is no definitive term used to describe CRCI across the literature, the following terms will be included: CRCI, cognitive impairment, dementia, mild cognitive impairment, cognitive dysfunction, and cognitive decline. The definition of cognitive impairment aligns with the American Psychological Association Dictionary of Psychology, which defines it as any impairment in perceptual, learning, memory, linguistic, or thinking abilities [21].

### Context

This qualitative synthesis will not place any restrictions on the context of patient experiences of CRCI and will include all geographical locations. Contexts are likely to include community-based settings such as the patient’s home, residential care facilities, or charitable organizations. Primary care settings may also be included such as general practitioner surgeries, pharmacies, or hospitals.

### Search Strategy

A literature search for qualitative and mixed methods studies will be conducted on the following web-based databases: PubMed, American Psychological Association PsycINFO, CINAHL, and Scopus. These databases were selected as they include a wide range of research fields deemed suitable to answer the objectives of this review. A librarian was consulted when forming the search terms to ensure no relevant terms were omitted. The search strategy has been adapted for each database to capture far-reaching and relevant studies. The specific search terms for each database are provided in Multimedia Appendix 1. The search terms were developed based on the PICo framework. The terms included the population of cancer survivors and the phenomenon of CRCI and its experience. There was no condition relating to context, therefore, there were no such terms included in the strategy.

### Data Extraction

The results from each database will be uploaded to Rayyan (Qatar Computing Research Institute), an artificial intelligence web-based tool for systematic reviews, where deduplication, collaborative title and abstract screening, collaborative full-text screening, and conflict resolution will be conducted. The remaining titles and abstracts will be screened to identify papers that adhere to the inclusion criteria. Screening will be performed by 2 independent reviewers; any uncertainty will be resolved by discussion. If necessary, a third reviewer will be requested to resolve disagreements. The included papers will then be retained for full-text screening. Subsequently, 2 reviewers will pilot the data extraction together by discussion, selecting 10% of the included studies to verify the agreement between reviewers. This will ensure that the data extracted will adhere to the PICo guidelines, specifically outlining the population, phenomenon of interest, and context relevant to the objective of this review. The included papers will be uploaded to NVivo (version 1.3; Lumivero) which will facilitate systematic and rigorous data synthesis [22]. The following data will be included in the data extraction table: author, year of publication, geographical location, design, data collection method, cancer type, impacted cognitive domains, management techniques, and possible themes. References will be stored in EndNote.

### Data Synthesis

The results section will be directly imported to NVivo for data synthesis. Data synthesis will be based on Thomas and Harden’s [23] 3-step thematic synthesis methodology. First, line-by-line coding will take place in the results section of each included study to interpret key concepts. These concepts surrounding patient experiences of CRCI will be translated from one study to another. Second, similar codes will be grouped to generate “descriptive themes.” These themes will be based on verbatim data from the selected studies. The codes and themes will be further reviewed to ensure that the data have been interpreted accurately. Finally**,** inductive reasoning will be used to determine “analytical themes” based on the descriptive themes to capture the patient experience of CRCI. These themes will directly relate objectives of this qualitative synthesis. The analytical themes will be based on the data interpretation of an independent reviewer.

### Critical Appraisal for Included Studies

Quality assessment of the included studies will be conducted using the Critical Appraisal Skills Programme (CASP) checklist tool, recommended for use in qualitative synthesis by Cochrane [24]. The CASP tool will be implemented by 2 independent reviewers to evaluate the quality of each of the included studies. Any potential disagreements between reviewers will be resolved verbally until a resolution is reached. An additional reviewer will be consulted if there is significant disagreement until the matter is resolved.

## Results

This qualitative synthesis will determine the current studies exploring the experience of CRCI across common cancers in adults aged 18 years or older. The results will presumably report on numerous cancer types such as breast cancer, prostate cancer, leukemia, and lung cancer. However, due to the heavy focus on breast cancer in the existing literature, this cancer type is expected to appear the most. The experiences of CRCI may be varied due to the broad definition of “experience” which could be used to describe many aspects of daily functioning. The management techniques adopted by the patients may also vary due to the different cognitive domains that may be impacted, as well as their employment status.

Although the inclusion criteria involve surveys, most of the qualitative data are likely to be derived from interviews and focus groups. Despite there being no evidence to suggest that CRCI only occurs after treatment, many of the current studies involve patients post treatment. Therefore, it is also reasonable to assume that the included studies will involve patients who have completed treatment rather than those during treatment. Despite reports of CRCI occurring during treatment, many papers report cases after treatment [25,26]. The results of this qualitative synthesis will be a novel addition to the literature and the results will clarify which cancer types and cognitive domains if any, are disproportionately impacted. We will also be able to determine the shared aspects of patients’ daily experiences of CRCI across common cancers.

## Discussion

### Principal Findings

To our knowledge, there is no other qualitative synthesis that explores the experience of CRCI across common cancer types. This qualitative synthesis will be one of a kind as it will collate the experiences of CRCI across cancer types and will identify common themes. From this, we can infer that the experience of CRCI is similar across all cancer types. If there are significant disparities across CRCI experiences, we can deduce which cancer types if any, are affected disproportionately. With global cancer survival rates rapidly increasing, it is essential to explore the long-lasting toxic impact of cancer treatments on neurological functioning. Patients need and are demanding effective CRCI interventions. This qualitative exploration will play a pivotal role in informing further developments of resources to support the maintenance and management of CRCI. There is vast literature surrounding cancer causes and treatment; however, this protocol will bridge the gap in the knowledge as it will provide useful insight into the collective experience of CRCI.

The results of this qualitative synthesis will be submitted for publishing in peer-reviewed scientific journals and will be included in a PhD thesis.

### Strengths and Limitations

The selected databases encompass a broad range of disciplines such as medicine, psychology, and nursing. The search strategy for this qualitative synthesis was defined with the assistance of an experienced librarian which allowed for comprehensive and systematic searching of the databases. A total of 2 reviewers will conduct systematic blind screening to decrease the risk of bias and ensure all relevant papers are included. A possible limitation of this qualitative synthesis is that papers were limited to the English language; therefore, some relevant papers may be omitted.

### Conclusion

This protocol summarizes the approach for a qualitative synthesis which aims to explore the experience of CRCI across common cancer types. With the rapid rise in global cancer survival, it is essential to explore life after cancer and the potential long-term toxic impact of cancer treatment on survivors’ neurological functioning and QoL. This qualitative synthesis will provide valuable insight into the lived experience of CRCI and the cognitive domains that may be disproportionately affected. There is growing demand for further management interventions and clinically tested treatments of CRCI and the qualitative exploration of the experience of CRCI is crucial for their development. This qualitative synthesis will inform future developments and will contribute to improving QoL after cancer.

## Appendix

Multimedia Appendix 1 Search strategy.

## Abbreviations

CASP: Critical Appraisal Skills Programme
CICI: chemotherapy-induced cognitive impairment
CRCI: cancer-related cognitive impairment
ENTREQ: Enhancing Transparency in Reporting the Synthesis of Qualitative Research
PICo: population phenomenon of interest and context
PRISMA: Preferred Reporting Items for Systematic Reviews and Meta-analyses
QoL: quality of life

## Data Availability

Data sharing is not applicable to this article as no data sets were generated or analyzed during this study.
