# Supplementary material for: The Experience of Cancer-Related Cognitive Impairment Across Common Cancers: Protocol for a Qualitative Systematic Review
Source: JMIR Res Protoc. 2024 May 31;13:e56888. doi: 10.2196/56888 (PMC11179027; doi:10.2196/56888)
Supplement: Multimedia Appendix 1 [file resprot_v13i1e56888_app1.docx]

**Appendix. Search Strategy**

PubMed

(neoplasm[MeSH Terms]) OR (cancer*[Title]) OR (“cancer survivor”[Title/Abstract]))

AND

((“cognitive dysfunction”[MeSH Terms]) OR (“cognitive impairment”[Title/Abstract]) OR (“cognitive decline” [Title/Abstract]”) OR (“cognitive complaints” [Title/Abstract]) OR (“chemotherapy-related cognitive impairment”[MeSH Terms]) OR (chemotherapy-related cognitive dysfunction”[Title/Abstract]) OR (“chemotherapy induced cognitive impairment” [Title/Abstract]) OR (“cancer-related cognitive impairment” [Title/Abstract]) OR (“chemo brain” [Title/Abstract]) OR (“chemo fog” [Title/Abstract]))

Filters:

- Year -2013-2023
- Language- English
- Species- Human

SCOPUS

ABS(cancer*) OR ABS(neoplasm*)

AND

ABS(“cancer related cognitive impairment”) OR ABS(chemobrain) OR ABS(chemofog) OR ABS(“cognitive impairment”)

Filters:

- Year -2013-2023
- Language- English
- Keyword - Human
- Document type - Article
- Source type - Journal

CINAHL

TI “cancer survivors”

AND

TI “Chemotherapy-related cognitive impairment” OR AB “Chemotherapy- related cognitive impairment” OR AB “Cognitive impairment OR AB “Cancer-related cognitive impairment”

AND

AB “Daily living functions” OR AB “employment” OR AB“quality of life”

Filters:

- Year -2013-2023
- Language- English
- Article type - Academic journals

APA PsycInfo

TI (cancer survivors OR survivors of cancer OR cancer survivorship)

OR

TI (neoplasms OR oncology OR cancer OR tumor OR malignancy)

AND

AB (chemobrain OR chemotherapy-related cognitive impairment OR chemotherapy induced cognitive impairment OR cancer related cognitive impairment)

OR

TI (chemobrain OR chemotherapy-related cognitive impairment OR chemotherapy induced cognitive impairment OR cancer related cognitive impairment)

AND

AB (work experience or job experience or employment experience)

Filters:

- Year -2013-2023
- Language- English
- Article type - Academic journals
